# The Effect of Terra-Cortril as Local Pain Medication on the Healing Process of a Fresh Extraction Socket: A Retrospective Cohort Study

**DOI:** 10.3390/jcm12134372

**Published:** 2023-06-29

**Authors:** Fauve Vuylsteke, Jan Cosyn, Manon Tytgat, Aryan Eghbali

**Affiliations:** 1 Department of Periodontology and Oral Implantology, Faculty of Medicine and Health Sciences, Oral Health Sciences, Ghent University, C. Heymanslaan 10, B-9000 Ghent, Belgium; jan.cosyn@ugent.be (J.C.); manon.tytgat@ugent.be (M.T.); 2 Private Practice Orthoparocare, Mankevosstraat 5, B-1860 Meise, Belgium; hoemane@hotmail.com

**Keywords:** Terra-Cortril, pain medication, extraction socket, wound healing, socket healing, implant

## Abstract

(1) Background: Use of Terra-Cortril may reduce pain and discomfort after tooth extraction. It is widely used among dentists, especially for third molar extraction and for treatment of dry socket. Only few case reports described unsuccessful healing with formation of myospherulosis. (2) Aim: The primary objective of this retrospective cohort study was to compare the occurrence of unsuccessful healing between extraction sockets locally treated with Terra-Cortril (TC) (exposure cohort) and extraction sockets subjected to unassisted healing (non-exposure cohort). The assessment of the three-dimensional morphology of the bone was a secondary objective. (3) Material and methods: The records of patients who had one or more extractions between 1 January 2020 and 1 July 2021 followed by implant placement in one private practice were retrieved and data were extracted. At the time of implant placement, practitioners established, both clinically and radiographically, if the healing was successful or unsuccessful. Explanatory demographic as well as clinical variables were extracted from patient files, checked and supplemented by contacting patients in cases of missing data. (4) Results: 69 patients were included who had, in total, 99 extractions. The exposure cohort included 55 sites, while the non-exposure cohort included 44 sites. A total of 56 sites demonstrated successful healing, and 53 showed unsuccessful healing. The regression model identified TC as a significant predictor (*p* = 0.014) for unsuccessful healing with an odds ratio of 2.86. Sites treated with Terra-Cortril had a significantly greater bone defect at level −1 mm, level −3 mm, and level −5 mm from the bone crest, and significantly greater maximal vertical and horizontal bone defects. At sites where Terra-Cortril was used, significantly more surgical consequences were reported (70.91% vs. 18.18%, *p* < 0.001). (5) Conclusions: fresh extraction sockets treated with Terra-Cortril increased the likelihood of unsuccessful healing with an odds ratio of 2.86. The greater defect size leads to significantly more limitations when planning implants at sites previously treated with TC.

## 1. Introduction

Terra-Cortril (TC) is an anti-inflammatory and anti-bacterial ointment that contains a combination of hydrocortisone, oxytetracycline and polymyxin B. Next to the traditional indication as primarily described by the manufacturers for ear and eye infections, oral surgeons and dentists have utilized TC in the past decades as a local analgesic after tooth extractions, to prevent and treat alveolar osteitis (AO), also known as dry socket [1].

Besides dry socket, which is a painful condition that follows the dissolution of the blood clot, other post extraction infectious complications related to third molar extraction can occur such as abscess, pain, fever, swelling and trismus. The overall incidence of infections is relatively low [2,3,4]. However, antibiotics are frequently prescribed prophylactically, especially in cases of complex surgical extractions and/or in patients with immunodeficient or systemic conditions such as HIV, diabetes and cancer [5]. This is in line with a recent systematic review by Lodi et al. They concluded that the use of antibiotics may reduce the risk of postsurgical infectious complications in patients undergoing third molar extractions by approximately 66% [6].

Other promising less invasive techniques such as coronectomy have been introduced to reduce the risk of complications related to third molar extraction. In systematic reviews, coronectomy was associated with a low incidence of complications in terms of inferior alveolar nerve injury (0–9.5%), lingual nerve injury (0–2%), postoperative pain (1.1–41.0%), swelling (4.6%), dry socket infection (2–12%), infection rate (1–9.5%) and pulp disease (0.9%). Migration of the retained roots seems to be a frequent occurrence (2–85.3%). Based on these findings, the authors concluded that coronectomy is a safe procedure, at least in the short term. Therefore, it can be indicated for teeth that are very close to the inferior alveolar nerve [7,8]. The study of Cosola et al. with a medium-term follow-up of 4 years strongly confirms the safety of this procedure [9].

Alveolar osteitis is a complication that occurs in 3–4% of routine dental extractions and in 25–30% of third molar extractions [10]. Topical application of a petroleum-based combination of tetracycline and hydrocortisone can reduce the occurrence of AO and post-operative pain [11,12,13]. However, some studies showed that there are side effects to this ointment [11,14,15]. 

In a case report of Fisher et al. inflammatory, granulomatous lesions were found after application of petroleum-based antibiotics in the extraction alveolus of third molars [16]. These lesions are described as myospherulosis because they consist of inflamed fibromuscular connective tissue with cystic spaces filled with spherules. Identical findings of myospherulosis have been shown in other case reports [17,18,19,20]. As previously found in an animal study [21], application of a 3% tetracycline ointment on connective tissue resulted in severe inflammation with necrotic cells and numerous polymorphonuclear leukocytes being present.

Rosai evaluated these findings in vitro by combining human erythrocytes with a 3% tetracycline ointment. The study found that the spherules were altered erythrocytes due to the petroleum and that the brown-black colour was caused by haemoglobin decomposition [22]. 

Besides the inevitable natural physiological process of tissue remodelling after tooth loss resulting in alveolar resorption, the occurrence of incomplete healing could result in even more volumetric tissue loss [23,24,25]. In addition, several factors such as necrosis, previous periodontal disease, presence of infection and traumatic injury, may further worsen this natural process [26]. As a result, this tissue loss may have a major impact on the clinical, radiographical and especially the aesthetic outcome. This additional tissue loss may even jeopardize any form of tooth replacement by means of dental implants since a sufficient vertical and horizontal volume of hard and soft tissue is required at the site of implantation [27].

To reduce the natural tissue loss to an acceptable level, several alveolar ridge preservation (ARP) techniques have been described in the literature, including guided bone regeneration (GBR), socket fillers, socket sealing and socket shield technique [28,29,30]. Although the main objective of these techniques is tissue preservation, less is known about their impact on post-operative pain and discomfort. When comparing ARP to natural healing, no significant difference was found in postoperative discomfort. However, ARP with a flapless approach resulted in significantly less postoperative pain than a flapped approach [31,32]. In contrast to ARP, the use of TC mainly focuses on pain relief and less on tissue preservation [13,33]. Available studies on the use of TC—although based on limited data of case reports—align with the findings of some periodontists working in a private practice, who have been treating patients with implants after TC had been inserted. At the time of tooth extraction, TC was applied on a haemostatic gelatin sponge. At implant placement, they repeatedly reported unsuccessful healing of the alveolus, both on clinical (visual incomplete bone formation at the site of implant placement with encapsulated black, tar-like substance after muco-periosteal flap elevation) and three-dimensional radiographic (cone-beam computed tomography = CBCT) examination. The question, therefore, arises whether the use of TC could interfere with the natural healing process of an extraction site and result in unsuccessful healing.

To the best of our knowledge, scientific data on the impact of TC on future implant sites have not been reported. Hence, the primary objective of this retrospective study was to compare the occurrence of unsuccessful healing between extraction sockets locally treated with TC (exposure cohort) and extraction sockets subjected to unassisted healing (non-exposure cohort). The assessment of the three-dimensional morphology of the bony defects, the clinical consequences for implant placement and implant survival were secondary objectives.

## 2. Materials and Methods

### 2.1. Study Design

The study was designed as a retrospective cohort study and was concluded in accordance with the Helsinki declaration of 1975 as revised in 2000. The study protocol was approved by the ethical committee of Ghent University Hospital (THE-2022-0273) 23 December 2022.

### 2.2. Patient Selection

The records of patients who had one or more extractions between 1 January 2020 and 1 July 2021 followed by implant placement in one private practice were retrieved and data were collected. After 1 July 2021, the use of TC in this clinic was discouraged. The dental implants were installed by two experienced surgeons.

For inclusion of cases in the study, CBCT scans taken before implant placement at minimally eighty days post extraction had to be available. Medical and demographic data were extracted from the patients’ records and patients were called to either confirm or collect any missing data. All patients needed one or more implants as tooth replacement. Extraction sites were divided in one of the following cohorts:

(1) “Exposure cohort” if TC had been administered;
(2) “Non-exposure cohort” if no TC had been administered.

The exclusion criteria were as follows:

(1) Tooth extraction in other practice;
(2) Other systemic antibiotics taken or topical antibiotics applied post-extraction;
(3) Smoking or use of any medication that could interfere with the healing process [34,35].

**Figure 1:**
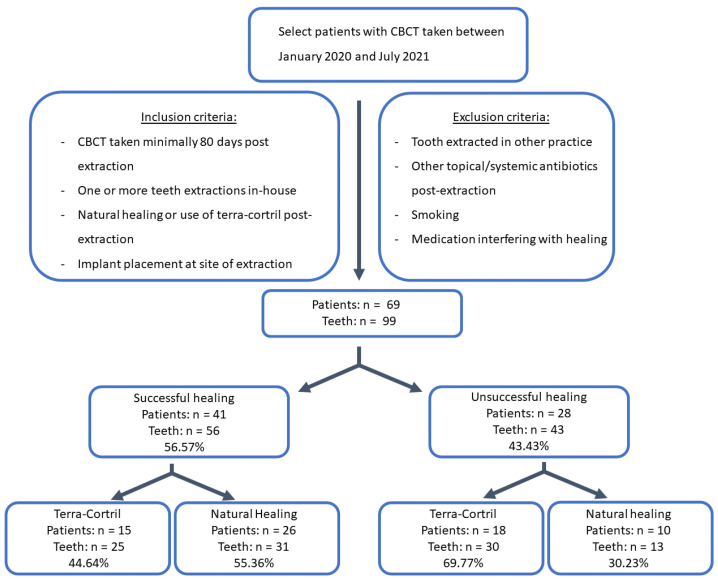
Flowchart of selection procedure.

The inclusion and exclusion criteria are illustrated in Figure 1.

### 2.3. Healing Outcome

Patient records were also scrutinized for the outcome (successful healing or unsuccessful healing). Successful healing was defined as complete bone fill of the alveolar socket defined by radiographic (CBCT) and clinical findings. 

**Figure 2:**
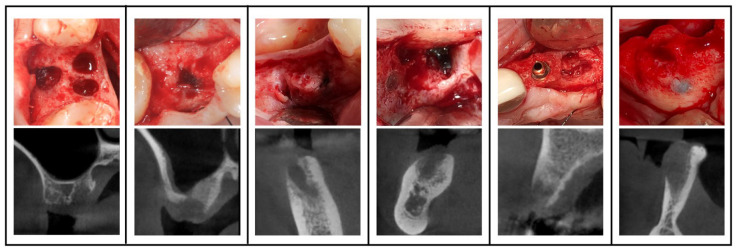
Clinical view of 5 cases with incomplete healing with black, tar-like substance in the socket and corresponding CBCT cross-sectional image (see pictures below) after the use of Terra-Cortril.

Unsuccessful healing was defined as incomplete bone fill of the alveolar socket (observed on the CBCT as remarkably more radiolucency of the alveolus compared with the surrounding bone) and clinically confirmed in one or more of the following ways:

- Deficient healing noted in the file by the operating dentist;
- Confirmed after opening the flap and observing an open alveolus with black, tar-like substance, as seen in Figure 2;
- Constrainedly placing the implant differently than planned due to a lack of primary stability or additional need of guided bone regeneration.

### 2.4. Explanatory Variables

The demographic and clinical variables were age, gender, apical radiolucency, jaw, tooth location (molar, premolar, anterior), treatment group (exposure versus non-exposure), and horizontal and vertical dimensions of the bony defect.

**Figure 3:**
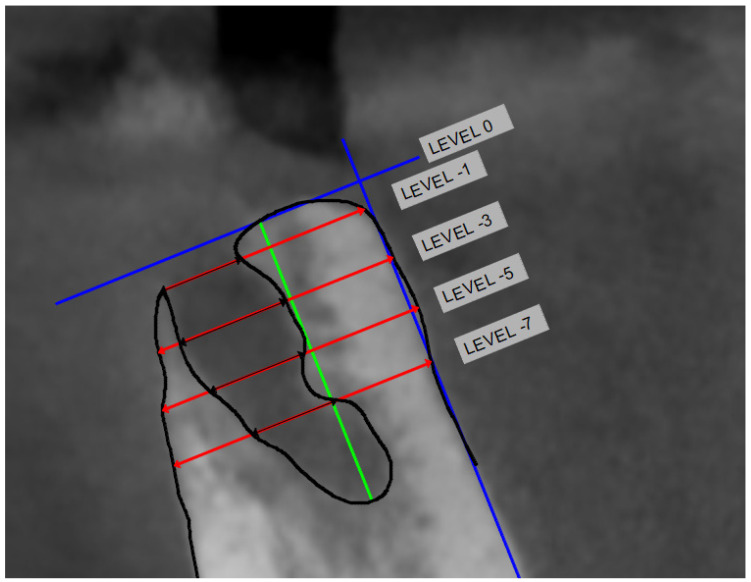
The cross-sectional CBCT slide pertaining to the center of the extracted root(s) were selected for measurement. First, most coronal bone was identified with a line perpendicular to the long axis of the lingual bone (blue line). Then, 4 lines were constructed perpendicular to this axis at the following levels: −1 mm apical to the most coronal bone line (L1), −3 mm apical to the most coronal bone line (L3), −5 mm apical to the most coronal bone line (L5), and −7 mm apical to the most coronal bone line (L7). Vmax is the distance from L0 to the most apical part of the defect (green line).

The horizontal and vertical dimensions of the bony defect (BD) were measured on the CBCT [36,37]. Horizontal bony defects were measured at levels −1 (L1), −3 (L3), −5 (L5) and −7 mm (L7) from the marginal bone of the socket (L0) as a percentage of bone defect, and as the greatest horizontal defect on the corresponding level (Hmax). Vertical loss was measured as the greatest vertical defect from marginal to apical of the socket (Vmax) (Figure 3).

Clinical consequences arising from this bony defect were processed and failure rates were calculated. Possible consequences were:

1. Prolonged healing time;
2. Two-phase placement;
3. Deviated angle of placement or position;
4. Additional need and use of guided bone regeneration (GBR) before or during implant placement.

An implant was considered as a failure when the records reported no integration, mobility, fracture, and/or any infection leading to implant removal. 

### 2.5. Statistical Analysis

Data analysis was performed in IBM SPSS 28 (SPSS Inc., Chicago, IL, USA) at site level. Continuous variables are presented as means and 95% confidence intervals (C.I.), while categorical variables were expressed as number of cases (n), percentages and odds ratio (OR). The associations between continuous variables and healing were analysed by simple binary logistic regression, while the associations between categorical variables and healing were analysed with the Chi-square test (χ^2^). The level of significance was set at 0.05.

A multiple binary logistic regression model was used to test the association between relevant variables and healing outcome, adjusting for confounding variables. 

The association between alveolar defect values and categorical variables was analysed with the Mann–Whitney U test and Kruskal–Wallis test. Finally, the association between different consequences and the use of TC was assessed using a multiple binary logistic.

## 3. Results

Seventy-five patients could be identified, of whom five were excluded because of smoking and one because of the use of bisphosphonates. Hence, 69 patients were included, with a total of 99 tooth extractions. The exposure cohort included 55 sites, while the non-exposure cohort included 44 sites. Fifty-six sites demonstrated successful healing and 43 showed unsuccessful healing. 

**Figure 4:**
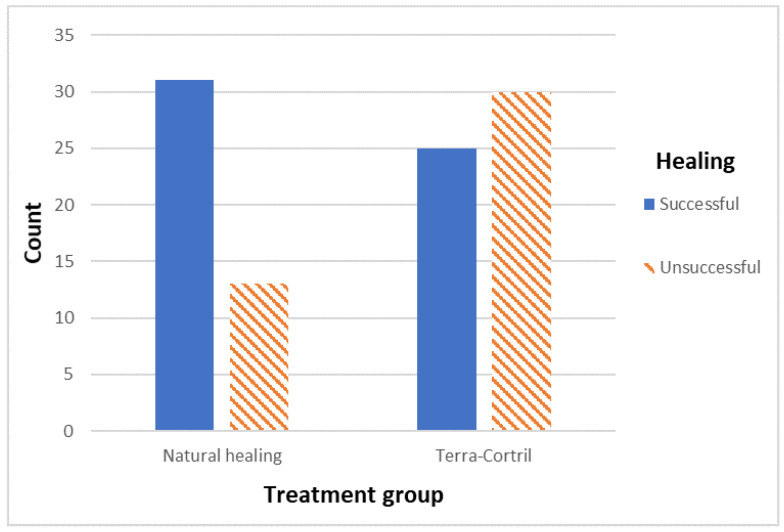
Bar chart visualizing TC vs. healing outcome.

Table 1 shows the demographic and clinical information of the categorical variables (gender, TC, jaw, apical radiolucency and tooth location) and the continuous variable (age at implant placement). Univariate analysis not correcting for confounding suggests that only treatment group (*p* = 0.013) and jaw (*p* = 0.023) were significantly associated with healing, while gender (*p* = 0.486), tooth location (*p* = 0.303) and age (*p* = 0.136) were not. The odds for unsuccessful healing were 2.86 times higher when TC had been used and 2.55 times higher in the lower jaw (Figure 4). 

A multiple binary logistic regression analysis correcting for confounding was performed to determine the association between potential factors and the healing outcome. The results of the final adjusted model only include the treatment group (*p* = 0.014) as a significant predictor of the healing outcome, increasing unsuccessful healing with an OR of 2.86 when TC was used.

The Mann–Whitney-U test and Kruskal–Wallis test were used to evaluate the mean alveolar defect size for all categorical variables (see Table 2). The defect size was significantly greater (*p* < 0.05) at all levels, apart from L7, when TC was used. There was also a significant greater defect size in the lower jaw at L5, L7 and Vmax (*p* < 0.05).

Several consequences on healing were logged and compared between patients with and without TC use. No consequences were observed in 81.82 % of the patients with natural healing, but only in 29.09% of the patients where TC was used. A multiple binary logistic regression analysis correcting for confounding was performed to determine the association between the potential factors and the consequence outcome. The results of the final adjusted model only included treatment group (*p* < 0.001) as significant predictor of the presence of consequences. A separate multiple binary logistic regression analysis was performed to determine the associations between potential factors and each of the possible consequences. Possible consequences following natural healing vs. TC were a longer healing time (2.27% vs. 1.82%—*p* = 0.596), a two-staged approach (0% vs. 32.73%—*p* = 0.997), pre-implant GBR (4.55% vs. 14.55%—*p* = 0.334), GBR at the time of implant placement (9.09% vs. 18.18%—*p* = 0.174) and a change of inclination or position (2.27% vs. 5.45%—*p* = 0.998). In some patients, multiple consequences were observed. Both groups had an implant survival of 100%. 

## 4. Discussion

The objective of the present retrospective cohort study was to investigate the effect of TC on the healing process of a fresh extraction socket. Given the limited knowledge on this effect, the results of the present study may add relevant clinical and radiographic information. To the best of our knowledge, no large clinical studies on the effect of TC on the healing of extraction sites have been carried out. However, there were some independent observations of lesions that could be traced back to the use of lipid-based antibiotic ointments [20,38,39,40]. Unfortunately, the majority of these studies were case reports, which makes it difficult to identify conclusive evidence and develop clinical guidelines. The lack of the latter makes it possible that TC is still used worldwide by dentists and oral surgeons without considering its possible side effects and, while asymptomatic, their clinical consequences. Furthermore, prospective studies do not exist, probably due to ethical restrictions with respect to the use of TC on a large population. 

Clearly, TC has a negative influence on the healing process of the extraction socket, as the results of the present study showed not only increased odds of incomplete healing (OR = 2.86), but also an impact on implant placement. A higher rate of clinical consequences was observed in the exposure cohort, that is, at the sites where TC had been used (*p* < 0.001), showing its inferior healing process. This includes (1) a longer healing time, (2) a two-stage protocol, (3) pre-implant GBR, (4) simultaneous GBR, and (5) a forced change in inclination or position of the implant. These findings are in line with the limited available case reports. Although none of the previous case reports were implant cases, one of the case reports described a change in the location of harvesting a block graft due to insufficient bone quality [16]. Zuniga et al. and Aleman Navas et al. each described a case with induced neuralgia [14,41]. However, most of the case reports may be considered coincidental findings as they are asymptomatic [17,18]. 

As most of these consequences were time related, resulting in a longer healing and treatment time, the additional use of the GBR techniques requires the use of biomaterials such as xenografts and membranes. The application of these biomaterials has financial impacts, which not only affect the patient but also the clinician [42]. From the clinician’s point of view, more surgical skills are required since surgery becomes more invasive, with vertical releasing incisions and periosteal elevation often inevitable. Moreover, invasive surgery may result in more scar tissue [43,44]. What is also important is the increased post-operative discomfort, such as swelling, hematoma and pain, which highly contrasts with the initial treatment objective of TC. 

The typical lesions, as seen in these patients, have been occasionally reported in the literature. Myospherulosis was first discovered by McClatchie et al. where Kenyan patients showed nodules on various places over the body, which could not be identified as any symptom of a known bacterial, viral, or other disease. Histological evaluation showed an increase in fibrous tissue and inflammatory infiltrate. Furthermore, it showed that the tissue was filled with cysts containing spherules, surrounded by histiocytes forming giant cells [45]. Following this report, multiple cases have come to light, including some in dentistry, and a clearer view has formed about what could be the cause of these lesions. On first sight, it seemed as if the cysts were formed by an unknown fungus in the ointment due to their aspect resembling a ‘bag of marbles’ [46]. Further histological evaluation could not determine any fungal origin [47]. Rosai noticed the resemblance to erythrocytes and discovered that the ointment altered erythrocytes, hence the spherules [22]. With these findings, questions also arose about the cause of these alterations.

Eslami et al. investigated the composition: the ointment carrier (petroleum jelly 90%, lanolin 10%) with and without tetracycline were inserted subcutaneously. Both histological results were similar to the histology of myospherulosis, with a more severe reaction for the ointment with tetracycline [21]. This demonstrates that not only the petroleum base, but also tetracycline itself has an adverse reaction on the connective tissue or at least intensifies the reaction of the carrier. In 1962, Boyne and Kruger found undissolved tetracycline fragments after placing cones with 5.0 mg oxytetracycline in the extraction wounds of dogs. Histological evaluation showed chronic inflammation of the surrounding connective tissue [48]. Apparently, undissolved fragments or remnants might interfere with the healing process. This observation is even observed using any type of ARP intervention and substitutes [49]. Hence, marked differences in outcomes in relation to the quality and the quantity of the regenerated tissue have been reported, as the remnants of the grafts often interfere with the normal healing process [50,51,52,53]. Other histological studies confirm these findings, showing that the quality of new tissue in the socket varies widely [50,54,55,56]. 

Besides the side effects, the use of TC could have benefits in some indications. The few clinical trials evaluating the effect of tetracycline-based ointments showed significantly less alveolar osteitis. It should be underlined that these trials only analysed patient-reported outcomes without any clinical or radiographic confirmation. Even if it can be assumed that tetracycline ointments do relieve pain, due to the asymptomatic appearance of most myospherulosis cases, the real consequences in terms of alveolar healing were not evaluated and probably underestimated [12,33,57,58]. This is in line with the findings of the present study where all the patients were asymptomatic and the impaired healing was noticed at the time of implant planning and confirmed during implant surgery. On the other hand, radiographic confirmation can only be registered after a healing process of three months. Julius et al. evaluated the extraction socket at the time of suture removal only if continuous pain was reported [12]. Obviously, pain perception, definition and tolerance vary between individuals and remain subjective. In addition, pain perception differs between patients and physicians [59]. The double check (clinical and radiographical) on the healing can be noted as a strength of this study. Furthermore, the periodontologists in the practice took a CBCT before implant placement by default, which limits selection bias. 

Several limitations should be considered when interpreting the results of the present study. First, this was a retrospective cohort study. In these types of studies, cause–effect relationships cannot be established due to a high risk of bias. The sample size was limited to the available data. It should be noted that the sample size is too small to draw definite conclusions and should be interpreted cautiously. On the other hand, as already mentioned and with the knowledge that we have, investigating this question in a prospective study would not be ethically justified as it could jeopardise a future implant site. Second, there were no CBCT scans provided before extraction, so we can only assess the remaining bony defect after healing and not the variations in healing of the alveolar ridge itself. The baseline ridge characteristics could influence the overall ridge dimensions after healing. However, a CBCT before extraction would have caused additional radiation. Moreover, methodological bias does exist as different dentists performed the extractions and applied TC according to preference. Finally, two periodontists assessed implant placement. Although all the dentists had at least five years of experience, the way of performing these treatments might have had an impact on the healing process, data registration and collection. This may be the most important source of bias. Furthermore, the definition of successful and unsuccessful healing process might result in some disparities in the outcomes. For this reason, in this study, both radiographic (CBCT) and the clinical findings of experienced clinicians were combined to describe these features. 

Not only with the results of this study in mind, but also considering the increased prevalence of microbial resistance and possible systemic side effects, local as well as systemic use of antibiotics should be avoided [60,61]. To eliminate risk factors of alveolar osteitis, dentists should refine their extraction technique to be as atraumatic as possible, and patients should be encouraged to initiate behavioral changes to address factors as poor oral hygiene and smoking [62]. Further research should be conducted to investigate other options for optimal alveolar healing which are already showing promising results. These include platelet rich fibrin, different rinsing agents, topical gel and low-level laser therapy since there is no consensus yet on the best way to prevent alveolar osteitis [63,64,65,66,67,68,69,70].

## 5. Conclusions

In conclusion, this retrospective cohort study demonstrated that the odds for unsuccessful healing were 2.86 times higher for fresh extraction sockets than those treated with TC. There were significantly greater defect sizes at L1, L3, L5, Hmax and Vmax for TC-treated sites than for sites subjected to natural healing, which leads to significantly more limitations when planning implants. Therefore, the use of lipid-based antibiotic ointments in extraction sockets should be avoided at all times.

## Figures and Tables

**Table 1:** Demographic and clinical information of categorical variables.

	Healing						
	Successful		Unsuccessful		OR		*p*-Value *
**Variable**	n	%	n	%			
**Gender**							
Male	26	53.06	23	46.94	0.75		0.486
Female	30	60.00	20	40.00			
**Treatment group**							
Natural healing	31	70.45	13	29.55	2.86		0.013
TC	25	45.45	30	54.55			
**Jaw**							
Maxilla	35	67.31	17	32.69	2.55		0.023
Mandible	21	44.68	26	55.32			
**Apical radiolucency**							
Yes	37	52.11	34	47.89	0.44		0.117
No	15	71.43	6	28.57			
**Tooth location**							
Anterior	16	61.54	10	38.46	0.98 **		0.303
Molar	26	61.90	16	38.10	1.94 ***		
Premolar	14	45.16	17	54.84			
	**Successful**		**Unsuccessful**		B	**df**	*p*-value ****
**Variable**	mean	95% CI	mean	95% CI			
**Age**							
	60.22	56.67–63.77	56.02	51.85–60.18	−0.023	1	0.136

Notes: OR, odds ratio; TC, Terra-Cortril. * Chi-square test; ** molar vs. anterior; *** premolar vs. anterior; **** simple binary logistic regression.

**Table 2:** Mean BD and 95% confidence interval (C.I.) for defect outcomes for all categorical variables. *p*-value determined with Mann–Whitney U-test or Kruskal–Wallis test (tooth location).

	L1			L3			L5			L7			Vmax			Hmax		
	Mean (%)	95% C.I.	*p*	Mean (%)	95% C.I.	*p*	Mean (%)	95% C.I.	*p*	Mean (%)	95% C.I.	*p*	Mean (mm)	95% C.I.	*p*	Mean (mm)	95% C.I.	*p*
**Treatment group**																		
Natural healing	21.99	11.20–32.79	0.011	17.52	7.89–27.16	0.009	14.00	5.41–22.59	0.006	12.22	4.19–20.25	0.072	3.16	1.68–4.65	0.030	2.01	1.16–2.86	**0.005**
TC	45.83	35.86–55.79		39.40	30.58–48.22		30.74	22.67–38.81		18.64	11.75–25.52		4.61	3.52–5.70		3.85	2.99–4.70	
**Jaw**																		
Lower jaw	39.25	28.27–50.22	0.245	35.00	24.90–45.09	0.073	30.86	21.95–39.76	0.011	21.63	13.40–29.87	0.015	5.02	3.68–6.35	0.022	3.59	2.67–4.51	0.098
Upper jaw	32.63	21.78–43.48		25.85	16.54–35.17		17.31	9.21–25.42		10.90	4.48–17.31		3.02	1.85–4.19		2.53	1.67–3.38	
**Gender**																		
Male	35.46	24.17–46.75	0.982	29.51	19.76–39.25	0.899	23.93	15.36–32.51	0.796	17.42	9.12–25.72	0.673	4.14	2.88–5.40	0.604	3.36	2.39–4.32	0.354
Female	35.95	25.28–46.63		30.67	20.91–40.43		23.39	14.60–32.18		14.60	7.92–21.28		3.80	2.50–5.10		2.71	1.89–3.53	
**Apical radio-translucency**																		
Yes	37.68	29.17–46.19	0.439	30.98	23.52–38.44	0.591	24.18	17.44–30.93	0.662	16.39	10.39–22.39	0.822	4.19	3.11–5.28	0.193	3.20	2.45–3.94	0.172
No	29.11	11.15–47.07		27.24	10.28–44.20		21.82	7.19–36.45		14.31	3.35–25.26		2.85	0.93–4.77		2.18	0.79–3.58	
**Tooth location**																		
Anterior	27.81	11.92–43.70	0.314	25.69	11.72–39.65	0.571	20.17	7.36–32.99	0.328	13.85	2.80–24.90	0.618	2.53	1.00–4.07	0.137	2.30	0.93–3.68	0.105
Pre-molar	37.80	25.56–50.05		30.70	19.72–41.68		23.39	13.82–32.95		14.64	5.69–23.59		4.65	3.07–6.23		3.83	2.67–5.00	
Molar	39.89	26.77–53.00		33.29	21.31–45.27		27.10	16.37–37.84		18.15	9.88–26.43		4.25	2.82–5.69		2.89	2.00–3.78	

Notes: Horizontal bone loss on level −1 (L1), −3 (L3), −5 (L5) and −7 mm (L7) from the marginal bone of the socket; Vmax, the greatest vertical defect from marginal to apical of the socket; Hmax, the greatest horizontal defect on corresponding level; TC, Terra-Cortril.

## Data Availability

The data presented in this study are available on request from the corresponding author. The data are not publicly available due to privacy and ethical restrictions.
